# Optimization of spin-lock times for T_1ρ_ mapping of human knee cartilage with bi- and stretched-exponential models

**DOI:** 10.1038/s41598-022-21269-2

**Published:** 2022-10-07

**Authors:** Hector L. de Moura, Rajiv G. Menon, Marcelo V. W. Zibetti, Ravinder R. Regatte

**Affiliations:** Department of Radiology, Center for Biomedical Imaging, New York University Grossman School of Medicine, New York, NY USA

**Keywords:** Scientific data, Cartilage

## Abstract

Two optimization criteria based on Cramér-Rao Bounds are compared between each other and with non-optimized schedules for T_1ρ_ mapping using synthetic data, model phantoms, and in-vivo knee cartilage. The curve fitting is done on complex-valued data using an iterative Nonlinear Least Squares (NLS) approach. The optimization criteria are compared based on the Mean Normalized Absolute Error (MNAE) and variance of the estimated parameters. The optimized spin-lock time (TSL) schedules provided improved results over the non-optimized schedules for all cases that were tested. The simulations showed that optimized schedules can reach the same precision and reduce acquisition times by 16.5 min (42%) for the bi-exponential model, and 6.6 min (22%) for the stretched-exponential model. In the model phantoms experiments, the bi-exponential MNAE was reduced from 0.47 to 0.36, while stretched-exponential from 0.28 to 0.20 with a Modified Cramér-Rao Lower Bound (MCRLB). In-vivo knee cartilage experiments show a reduction in bi-exponential MNAE from 0.47 to 0.31, and stretched-exponential from 0.047 to 0.039. The optimized spin-lock times criteria reduced the error in all cases, being more significant in the synthetic data and model phantoms. The optimized TSL schedules can be either used to improve the quality of parameter maps or reduce scan time.

## Introduction

Osteoarthritis (OA) is a degenerative disease that causes degradation and loss of articular cartilage. Its symptoms include pain, stiffness, and swelling in the knee joint^1^. These symptoms only appear after irreparable damage has occurred^2^. The early detection of OA can help to prevent these symptoms and mitigate damage before the disease reaches an irreversible stage^3^. Among the many methods used to detect OA, MRI shows the most sensitivity^4,5^.

Articular cartilage is mainly composed of water (~ 75%), collagen (~ 20%), and proteoglycans (PGs, ~ 5%)^6,7^. In the early stages of OA, there is a breakdown of the collagen network, increasing water content, and loss of PGs^8^. The degree of decrease in PG content is associated with the severity of the disease^5,7^.

Among other parameters such as collagen and water content and orientation of the collagen fibers, the spin–lattice relaxation in the rotating frame (T_1ρ_) is also sensitive to PG content in articular cartilage. Previous studies demonstrated the relation between cartilage degradation and elevated T_1ρ_ relaxation times^5,9^. T_1ρ_ is also less variant to orientation than T_2_ depending on the spin-lock frequency^10^. T_1ρ_ maps can be produced by acquiring several T_1ρ_ -weighted images using different spin-lock times. Their accuracy is tied to the number of images acquired as well as their signal-to-noise ratios (SNR). Increasing the number of images will also increase the duration of the acquisition, which is undesirable as the chances of the subject’s motion increase. The SNR can be increased by averaging several images or using multiple receiver channels. The choice of spin-lock times (TSL) can also improve the accuracy of T_1ρ_ maps^11,12^. Optimizing the TSL schedules makes it possible to reduce acquisition time while obtaining parameter maps of the same quality.

The Cramér-Rao Lower Bound (CRLB) has been applied to sampling schedule optimization for T_1_ and T_2_ mapping^13–16^, and Magnetic Resonance Fingerprinting^17,18^. Optimizing the sampling schedule leads to better conditioning of the matrices used for Nonlinear Least Squares (NLS)^12^. A previous study by Zibetti et al.^12^ showed the efficacy of optimizing the sampling schedules using the CRLB and a modified CRLB (MCRLB). This reduced the error in mono-exponential T_1ρ_ fitting while using only 2 images, effectively reducing the acquisition time.

Previous studies reported non-monoexponential T_2_^19^ and T_1ρ_^20^ relaxation in bovine cartilage associated with collagen fiber orientation and three water compartments: water bound to collagen, water tightly bound to PG, and bulk water loosely bound to PG. These studies used multi-exponential models like bi-exponential and tri-exponential. Other studies^21,22^ also reported that this non-monoexponential T_2_ relaxation can be well fitted with the so-called stretched-exponential models.

More recently, Sharafi et al.^7^ reports similar non-monoexponential T_1ρ_ relaxation in human knee cartilage using the bi-exponential model. Non-monoexponential T_1ρ_ relaxation was also reported^23^ in intervertebral discs and a correlation between the stretched-exponential parameters and glycosaminoglycan (GAG) content variation. This could also provide better sensitivity for detecting early degeneration in knee cartilage.

Fitting these multi-exponential models requires more images than a monoexponential model, proportional to the number of parameters, resulting in longer acquisition times. Therefore, the optimization of sampling schedules is critical for these models. In this paper, we extend Zibetti et al.’s^12^ approach to the CRLB and MCRLB optimization of TSL sampling schedules for the bi-exponential and stretched-exponential models. Another difference from previous works is the use of different weights for each of the model’s parameters. We show that the optimized schedules can improve the performance of the fitting process for both multi-exponential models. This approach can also generate shorter schedules that can greatly reduce acquisition time while maintaining the quality of the fitting process of a longer, but non-optimized, schedule. The speedup can reach up to 1.7 times that of a non-optimized schedule. We demonstrate these results through simulated data, model phantoms, and in-vivo knee cartilage experiments.

## Methods

### Data acquisition and reconstruction

All the MRI scans were performed on a 3 T, whole-body clinical MRI scanner (Prisma, Siemens Healthcare, Erlangen, Germany) with a 15-channel transmit/receive knee coil (QED, Cleveland OH). The 3D-T1ρ-weighted datasets were acquired using a modified 3D Cartesian ultra-fast spoiled gradient echo (Turbo FLASH) sequence^12^ for variable TSL as shown in supporting information Fig. S1.

The protocol used is composed of a sequence of T_1ρ_ preparation modules P, 3D imaging modules A, and longitudinal magnetization restoration modules R. Module P uses a spin-lock frequency of 500 Hz and TSL according to the sampling schedule. Module A acquires 64 k-space lines (with 256 samples each) per preparation pulse, using a steady-state sequence with TR/TE = 7.63 ms/3.67 ms and flip angle of 8°, and a receiver bandwidth of 510 Hz/pixel. Longitudinal magnetization recovery module R, consisting of a delay of 1000 ms, is used after module A. The set of modules P-A-R is repeated 128 times to capture a data matrix of size 256 × 128 × 64 per TSL. The slice thickness is 2 mm and the FOVs are defined as 160 mm × 160 mm for the egg phantom and 196 mm × 196 mm for the in-vivo knee joint. After upsampling in phase direction, the resolutions are 0.6 mm × 0.6 mm × 2 mm and 0.76 mm × 0.76 mm × 2 mm for the egg phantom and knee joint respectively. Each module P takes approximately the same time as the TSL used, that is, between 0.5 and 55 ms, each module A takes approximately 64 × TR = 486.4 ms, and each module R takes 1000 ms. One set of modules makes up a shot, that takes approximately 1.5 s. To complete the data matrix, 128 shots, the total time takes between 3.17 and 3.28 min, depending on the TSL used.

The 2D slices are recovered using SENSE^24^, which solves:

1 $$\widehat{{{\varvec{x}}_{t} }} = \mathop {\text{arg min}}\limits_{{{\varvec{x}}_{t} }} {\varvec{y}}_{t} - {\varvec{FBx}}_{t2}^{2} ,$$

where ${\varvec{x}}_{t}$ is a complex-valued vector representing an image with TSL $t$ and size Ny × Nz = 128 × 64, with Ny being the image size on the y-axis and Nz the size on the z-axis. The vector ${\varvec{y}}_{t}$ represents the captured k-space data with size Ny × Nz × Nc, where Nc is the number of coils. The matrix ${\varvec{B}}$ contains the coil sensitivities and phase compensation, and ***F*** the Fourier transforms of all sensitivity-weighted images.

A non-optimized TSL schedule, composed of linearly spaced samples between 0.5 and 55 ms was used as the baseline for comparison against the optimized schedules. Monte Carlo simulations were carried out to assess the performance of the optimized schedules in controlled scenarios. The experiments were performed using a model phantom and human volunteers. The model phantom was composed of three pairs of unfertilized chicken eggs^25,26^ that were purchased from a local store. The eggs were used in three conditions: raw, soft-boiled, or hard-boiled. T_1ρ_ relaxation in eggs shows non-monoexponential behavior^26^, especially in the yolks. The human volunteers were 3 healthy males with a mean age of 37 ± 16 years.

This study was approved by the institutional review board (IRB) of New York University Langone Health and was compliant with the health insurance portability and accountability act (HIPAA). Every volunteer provided written informed consent after an explanation of the study and the protocol, and before scanning, as per IRB guidelines. All the methods reported herein were performed in accordance with the institutional guidelines and regulations.

Most figures were entirely generated by Matlab^27^ code, while Figs. 3, 4, 5 and 6 were partially generated by Matlab code and manually assembled to their final format using the vector graphics software Inkscape^28^.

### Bi- and stretched-exponential models

The use of bi- and stretched-exponential models can better characterize decays when the voxels might contain different compartments. The more commonly used of these models is the bi-exponential model. This model considers two compartments, one with short decay and the other with a long decay, each with a respective amplitude. It can be described as

2 $$s_{bi} \left( {t,{\varvec{n}}} \right) = A\left( {\varvec{n}} \right){ }\left[ {f\left( {\varvec{n}} \right){\text{exp}}\left( {\frac{ - t}{{{\text{ T}}_{{1{\rho s}}} \left( {\varvec{n}} \right)}}} \right){ } + { }\left( {1 - f\left( {\varvec{n}} \right)} \right){\text{exp}}\left( {\frac{ - t}{{{\text{T}}_{{1{\rho 1}}} \left( {\varvec{n}} \right)}}} \right)} \right] + { }\eta \left( {t,{\varvec{n}}} \right),$$

where $A\left( {\varvec{n}} \right)$ is the complex-valued signal amplitude at position ${\varvec{n}}$, $f\left( {\varvec{n}} \right)$ represents the real-valued short fraction, i.e. the contribution of the compartment with short decay to the voxel amplitude, ${\text{T}}_{{1{{\uprho{\rm s}}}}}$ denotes the shorter relaxation time, ${\text{T}}_{{1{{\uprho {\rm l}}}}} {\text{T}}_{{1{{\uprho {\rm l}}}}}$ denotes longer relaxation time, and $\eta \left( {t,{\varvec{n}}} \right)$ is a complex-valued white Gaussian noise.

Similarly, we define the second exponential model used, the stretched-exponential model. It can be defined as

3 $$s_{st} \left( {t,{\varvec{n}}} \right) = A\left( {\varvec{n}} \right){\text{ exp}}\left( { - \left( {\frac{t}{{{\text{T}}_{{1{\uprho }}} {*}\left( {\varvec{n}} \right)}}} \right)^{{\beta \left( {\varvec{n}} \right)}} } \right) + { }\eta \left( {t,{\varvec{n}}} \right),$$

where ${\text{T}}_{{1{\uprho }}} *$ denotes the characteristic relaxation time of the voxel, and $\beta$ is the stretching exponent in the range of $0 < \beta \le 1$. This model considers the decay as the sum of independently relaxing compartments within a voxel. Johnston et al.^29^ provided a detailed study of the model, including a unique physical interpretation of it. The stretching exponent parameter allows the modeling of a broad continuous distribution of relaxation times, representing varying degrees of microstructural complexity in the tissue, and was shown to be correlated with a decrease in water and GAG contents^23^. This correlation could be useful to detect early changes in knee cartilage composition. Essentially, as $\beta$ gets closer to 1, the more monoexponential the voxel is. Non-monoexponential voxels are shown as voxels with $\beta$ smaller than 1.

Although its interpretation is not as clear as the bi-exponential model, the stretched-exponential has three parameters and its optimization is more stable, as we will show later. As the models are the same for every position ${\varvec{n}}$ and all model parameters are position-dependent we will omit it from here on to keep the notation clear.

For the bi-exponential model, we can define the parameter vector $\varvec{\theta }_{{\user2{bi}}} = \left[ {{\text{A}},{\text{f}},{\text{~T}}_{{1{{\uprho {\rm s}}}}} ,{\text{~T}}_{{1{{\uprho {\rm l}}}}} } \right]$, for the stretched-exponential we define it as ${\varvec{\theta}}_{{{\varvec{st}}}} \user2{ } = \user2{ }\left[ {{\text{A}},{\text{T}}_{{1{\uprho }}} ,{\upbeta }\user2{ }} \right]$. The exponential signals are sampled at $K$ different TSLs defined by the sampling schedule ${\varvec{t}}$ and we can thus represent it by ${\varvec{s}}\left( {{\varvec{t}},{\varvec{\theta}}} \right) = \left[ {s\left( {t_{1} ,{\varvec{\theta}}} \right), s\left( {t_{2} ,{\varvec{\theta}}} \right),... ,s\left( {t_{K} ,{\varvec{\theta}}} \right)} \right]^{T}$. This expression emphasizes the dependence on both the sampling schedule ${\varvec{t}}$ and the model parameters ${\varvec{\theta}}$.

### Curve fitting algorithm

The curve fitting for both models is done using the Nonlinear Least Squares (NLS) described by

4 $$\hat{\user2{\theta }} = arg\mathop {\min }\limits_{{{\varvec{\theta}} \in {{\varvec{\Theta}}}}} \mathop \sum \limits_{k = 1}^{K} \left| {x\left( {t_{k} } \right) - s\left( {t_{k} ,{\varvec{\theta}}} \right)} \right|^{2} ,$$

where **Θ** represents the set of possible parameter values. The solution to Eq. (4) is found by using the Conjugate Gradient Steihaug’s trust-region (CGSTR) algorithm^30^. This iterative algorithm is stopped after 2500 iterations or when the normalized step is smaller than 10^–9^. The signal amplitude is normalized before fitting, so the normalization factor is included in parameter A. The set **Θ** contains every possible combination of the values inside the ranges displayed in Table 1. The values in Table 1 were chosen to represent the expected range of values in knee cartilage. In egg yolks, higher ${\text{T}}_{{1{\uprho }}}$ values can be expected for the long component^26^.

**Table 1:** Minimum and maximum values were considered for each of the models’ parameters. The estimated values are constrained to improve estimation stability.

Parameters	A(a.u.)	f	${T}_{1{\rho }_{s}}$[ms]	${T}_{1{\rho }_{l}}$[ms]	${T}_{1\rho }*$[ms]	β
Minimum	0	0.05	1	30	10	0.4
Maximum	1e10	0.95	10	90	90	1

Usually, the curve fitting is done with magnitude-only data, here we used the complex-valued data. Magnitude-only fitting must account for Rician distributed noise and also rely on data weighting and noise thresholding to improve performance^31^. Complex-valued estimators are statistically efficient^31^ and do not require these additional steps. Supporting information Fig. S2 illustrates the comparison between complex-valued and magnitude fitting. Simulated data for the bi- and stretched-exponential models were generated using values in the ranges shown in Table 1 and using different levels of SNR.

### Spin-lock time schedule optimization

The CRLB is a lower bound for the variance of unbiased estimators^32^. A previous study^12^ showed that using the CRLB criterium for the optimization of TSLs is very similar to the direct optimization of the estimator mean squared error using a matched sampling-fitting (MSF) approach. Similarly, minimizing the MCRLB lead to results similar to an MSF approach to minimizing the normalized mean absolute error. Even though our fitting method is constrained, this previous result shows that the CRLB for unbiased estimators is enough for our approach.

The CRLB is generalized to multi-parameter estimators by the Cramér-Rao Matrix (CRM), which contains the CRLB for each parameter in its main diagonal matrix. The CRM is defined as

5 $${\varvec{V}}\left( {{\varvec{t}},{\varvec{\theta}}} \right){ } = { }{\varvec{I}}^{ - 1} \left( {{\varvec{t}},{\varvec{\theta}}} \right),$$

where the Fisher Information Matrix (FIM), $I\left( {t,\theta } \right),$ is defined as

6 $${\varvec{I}}\left( {{\varvec{t}},{\varvec{\theta}}} \right){ } = { }{\mathbb{E}}\left[ {\left( {\frac{{\partial { }\ln { }p\left( {{\varvec{x}}\left( {{\varvec{t}},{\varvec{\theta}}} \right)} \right)}}{{\partial {\varvec{\theta}}}}} \right)\left( {\frac{{\partial { }\ln { }p\left( {{\varvec{x}}\left( {{\varvec{t}},{\varvec{\theta}}} \right)} \right)}}{{\partial {\varvec{\theta}}}}} \right)^{T} \left| {{\varvec{t}},{\varvec{\theta}}} \right.} \right],$$

in which $\ln p\left( {{\varvec{x}}\left( {{\varvec{t}},{\varvec{\theta}}} \right)} \right)$ is the natural logarithm of the probability density function given by Eq. (2) or (3). The FIM can also be obtained using the Jacobian of Eqs. (2) and (3), like

7 $${\varvec{I}}\left( {{\varvec{t}},{\varvec{\theta}}} \right){ } = { }\frac{1}{{\sigma_{\eta }^{2} }}\mathop \sum \limits_{k = 1}^{K} {\varvec{J}}\left( {t_{k} ,{\varvec{\theta}}} \right)^{H} { }{\varvec{J}}\left( {t_{k} ,{\varvec{\theta}}} \right).$$

ae row $k$ of the Jacobian matrix, denoted ${\varvec{J}}\left( {t_{k} ,{\varvec{\theta}}} \right)$, is dependent on the choice of TSL and also the expected distribution of parameters. For the bi-exponential model, it is an $1 \times 4$ vector defined as

8 $${\varvec{J}}_{bi} \left( {t_{k} ,{\varvec{\theta}}} \right) = \left[ {\begin{array}{*{20}l} {f {\text{exp}}\left( {\frac{{ - t_{k} }}{{{\text{ T}}_{{1{\rho s}}} }}} \right) + \left( {1 - f} \right) {\text{exp}}\left( {\frac{{ - t_{k} }}{{{\text{T}}_{{1{\rho 1}}} }}} \right)} \hfill \\ {A\left[ {{\text{exp}}\left( {\frac{{ - t_{k} }}{{{\text{ T}}_{{1{\rho s}}} }}} \right) - {\text{exp}}\left( {\frac{{ - t_{k} }}{{{\text{T}}_{{1{\rho 1}}} }}} \right)} \right]} \hfill \\ {\frac{{ - t_{k} \left( {A{ }f} \right)}}{{{\text{ T}}_{{1{\rho s}}}^{2} }} {\text{exp}}\left( {\frac{{ - t_{k} }}{{{\text{T}}_{{1{\rho s}}} }}} \right)} \hfill \\ {\frac{{ - t_{k} \left[ {A{ }\left( {1 - f} \right)} \right]}}{{{\text{ T}}_{{1{\rho 1}}}^{2} }} {\text{exp}}\left( {\frac{{ - t_{k} }}{{{\text{T}}_{{1{\rho 1}}} }}} \right)} \hfill \\ \end{array} } \right]^{T} ,$$

and for the stretched-exponential model, it is a $1 \times 3$ vector denoted as

9 $${\varvec{J}}_{st} \left( {t_{k} ,{\varvec{\theta}}} \right) = \left[ {\begin{array}{*{20}l} {{\text{exp}}\left( { - \left( {\frac{{t_{k} }}{{{\text{T}}_{{1{\uprho }}}^{*} }}} \right)^{\beta } } \right)} \hfill \\ {\frac{{A{ }\beta {\text{ exp}}\left( { - \left( {t_{k} /{\text{T}}_{{1{\uprho }}}^{*} } \right)^{\beta } } \right)}}{{{\text{T}}_{{1{\uprho }}}^{*} }}\left( {\frac{{t_{k} }}{{{\text{T}}_{{1{\uprho }}}^{*} }}} \right)^{\beta } } \hfill \\ { - A{\text{ exp}}\left( { - \left( {t_{k} /{\text{T}}_{{1{\uprho }}}^{*} } \right)^{\beta } } \right)\left( {\frac{{t_{k} }}{{{\text{T}}_{{1{\uprho }}}^{*} }}} \right)^{\beta } \log \left( {\frac{{t_{k} }}{{{\text{T}}_{{1{\uprho }}}^{*} }}} \right)} \hfill \\ \end{array} } \right]^{T} .$$

Both expressions are dependent on the parameters ${\varvec{\theta}}$, and so, the optimal sampling schedule will change according to the distribution of these parameters. To account for this, we first optimized considering a uniform distribution within the ranges shown in Table 1, the resulting schedules were used for the synthetic data experiments.

To obtain the optimal schedule, $\hat{\user2{t}},$ we look for the schedule that minimizes the combined CRLBs averaged over the parameters ${\varvec{\theta}}$, this criterium is defined as

10 $$\hat{\user2{t}} = {\text{arg}}\mathop {\min }\limits_{{{\varvec{t}} \in {\varvec{T}}}} \frac{1}{S}\mathop \sum \limits_{s = 1}^{S} \left( {\mathop \sum \limits_{i} w_{i} \left[ {{\varvec{V}}\left( {{\varvec{t}},{\varvec{\theta}}_{{\varvec{s}}} } \right)} \right]_{i,i} } \right),$$

where $w_{i}$ is the weight given to each parameter according to its importance, ${\varvec{\theta}}_{{\varvec{s}}}$ is the s-th sample drawn from the distribution of the parameters. Another possible criterium is the Modified CRLB^12^ (MCRLB), described by

11 $$\hat{\user2{t}} = {\text{ arg}}\mathop {\min }\limits_{{{\varvec{t}} \in {\varvec{T}}}} \frac{1}{S}\mathop \sum \limits_{s = 1}^{S} \left( {\mathop \sum \limits_{i} w_{i} \frac{{\sqrt {\left| {\left[ {{\varvec{V}}\left( {{\varvec{t}},{\varvec{\theta}}_{{\varvec{s}}} } \right)} \right]_{i,i} } \right|} }}{{\left| {\left[ {{\varvec{\theta}}_{s} } \right]_{i} } \right|}}} \right).$$

The MCRLB favors equal relative precision across the components and also avoids that large values in the CRM dominate the overall cost^12,15^. The weighting vectors for both models were determined, empirically, by searching the values that better equalized the errors of every parameter in each model. The idea is to verify which parameters have higher normalized bounds and increase their weighting so the algorithm prioritizes them. We repeated the optimization process with different weights until the normalized bounds were closer to each other. This led to ${\varvec{w}} = \left[ {0, 0.3, 0.4, 0.3} \right]$ for the bi-exponential, and ${\varvec{w}} = \left[ {0, 0.9, 0.1} \right]$ for the stretched-exponential model. Since the amplitude parameter is easier to estimate, its weight was zero for both models. While the bi-exponential parameter weights are similar to each other, the weights for the stretched-exponential were very different. That means the bound for the stretching parameter was already much lower when considering equal weights.

The possible values of TSLs, ${\varvec{t}}$, are defined over a grid before the optimization. For this study, we used a non-uniform grid starting from 0.5 to 5 ms, with 0.5 ms steps, and from 6 to 55 ms, with 1 ms steps. To optimize Eqs. (10) and (11), we used the Pareto Optimization for Subset Selection (POSS)^33^ algorithm. The decision to limit the grid to 55 ms comes from the SNR of the images obtained with longer TSLs. At 55 ms, the signal is already close to noise levels. Another detail here is that longer TSLs could lead to Specific Absorption Rates (SAR) above the Federal Drug Administration (FDA) recommended levels.

To validate the results, we compare the estimated values for each parameter against reference values. For the synthetic data, the reference values are known, but for model phantoms and in-vivo data, the reference values are estimated by fitting the corresponding model with every acquired TSL.

The comparison is done using the MNAE, defined as

12 $$MNAE_{i} { } = { }\frac{1}{{\left| {ROI} \right|}}\mathop \sum \limits_{{{\varvec{n}} \in { }ROI}} \frac{{\left| {\theta_{i} - \hat{\theta }_{i} } \right|}}{{\left| {\theta_{i} } \right|}},$$

to compare the parameters against the reference, where $\hat{\user2{\theta }}$ is the estimated parameter value and ${\varvec{\theta}}$ is the reference. To compare the schedules across different parameters we combine the NAE from all parameters as a weighted average defined by

13 $$Combined\,\, NAE = \frac{{\mathop \sum \nolimits_{i} w_{i} .\,MNAE_{i} }}{{\mathop \sum \nolimits_{i} w_{i} }},$$

which can be averaged across voxels for a single number comparison.

We also used the R-Squared (R^2^) metric to evaluate how well the estimated parameters fit the acquired data from model phantoms and human volunteers. The metric is defined as

14 $$R^{2} = \frac{{\mathop \sum \nolimits_{s} \left( {\hat{\theta }_{s} - \overline{\theta }} \right)^{2} }}{{\mathop \sum \nolimits_{s} \left( {\theta_{s} - \overline{\theta }} \right)^{2} }}{ }.$$

Lastly, we use the corrected Akaike Information Criteria (AICc) to determine which model best fits the data in small sampled studies^34,35^. Assuming all data points are independent and identically distributed with a normal distribution around the fitted curve, the AICc is defined as

15 $$AICc = 2P + K\log \left( \frac{SSE}{K} \right) + \frac{{2P\left( {P + 1} \right)}}{K - P - 1},$$

where $P$ denotes the number of model parameters, and SSE denotes the Sum of Squared Error of the fitted curve. The model with lower AICc is the one that better fits the data points, while accounting for the number of model parameters. We used this metric to determine the percentage of voxels that are better fitted with either the bi- or stretched-exponential models for the references.

## Results

### Evaluation with synthetic data with known ground truth

Monte Carlo simulations were carried out to evaluate the performance of both the CRLB and the MCRLB as optimization criteria against a non-optimized sampling schedule. The non-optimized schedule consists of linearly spaced timings in the same grid as the optimized ones.

The simulations consider several samples ${\varvec{\theta}}_{{\varvec{s}}}$, with which decay curves are synthesized and sampled, according to the evaluated schedule. The sampled signals are then corrupted with complex-valued white Gaussian noise so that it has an SNR = 30. Different schedules were evaluated using 4–12 TSLs for the bi-exponential model, and 3–9 for the stretched-exponential model. For each *K*, there are two optimized schedules and one non-optimized. The sampling schedules used are displayed in Table 2.

**Table 2:** Optimized and non-optimized sampling schedules for both multi-exponential models.

**Bi-exponential**		
**K**	**CRLB**	**MCRLB**
4	[0.5 4.7 22 55]	[0.5 3 20 55]
5	[0.5 4.7 23 55 55]	[0.5 2.1 6 22 55]
6	[0.5 5 24 24 55 55]	[0.5 2.2 6 21 22 55]
7	[0.5 5 24 25 55 55 55]	[0.5 2.1 6 23 23 55 55]
8	[0.5 5 25 25 25 55 55 55]	[0.5 1.7 5 6 22 22 55 55]
9	[0.5 4.4 7 24 24 24 55 55 55]	[0.5 1.8 6 6 22 22 22 55 55]
10	[0.5 4.4 7 24 25 25 55 55 55 55]	[0.5 1.8 6 6 23 23 23 55 55 55]
11	[0.5 4.6 7 24 24 25 25 55 55 55 55]	[0.5 1.8 6 6 22 23 23 23 55 55 55]
12	[0.5 4.6 7 25 25 25 25 55 55 55 55 55]	[0.5 1.8 6 6 23 23 23 23 55 55 55 55]
**Non-optimized**		
4	[0.5 18 37 55]	
5	[0.5 14 28 41 55]	
6	[0.5 11 22 33 44 55]	
7	[0.5 9 18 28 37 46 55]	
8	[0.5 8 16 24 31 39 47 55]	
9	[0.5 7 14 21 28 34 41 48 55]	
10	[0.5 6 12 18 24 31 37 43 49 55]	
11	[0.5 6 11 17 22 28 33 39 44 50 55]	
12	[0.5 5 10 15 20 25 30 35 40 45 50 55]	
**Stretched-exponential**		
	CRLB	MCRLB
3	[0.5 11 55]	[0.5 11 55]
4	[0.5 10 16 55]	[0.5 12 14 55]
5	[0.5 12 13 55 55]	[0.5 12 13 55 55]
6	[0.5 0.5 9 17 55 55]	[0.5 0.5 11 11 55 55]
7	[0.5 0.5 11 11 55 55 55]	[0.5 0.5 12 12 12 55 55]
8	[0.5 0.5 9 10 30 55 55 55]	[0.5 0.5 12 12 12 55 55 55]
9	[0.5 0.5 12 12 12 55 55 55 55]	[0.5 0.5 0.5 11 11 12 55 55 55]
**Non-optimized**		
3	[0.5 28 55]	
4	[0.5 19 37 55]	
5	[0.5 14 28 42 55]	
6	[0.5 12 23 33 44 55]	
7	[0.5 10 19 28 37 46 55]	
8	[0.5 9 16 24 32 40 47 55]	
9	[0.5 8 14 21 28 35 42 48 55]	

**Figure 1:**
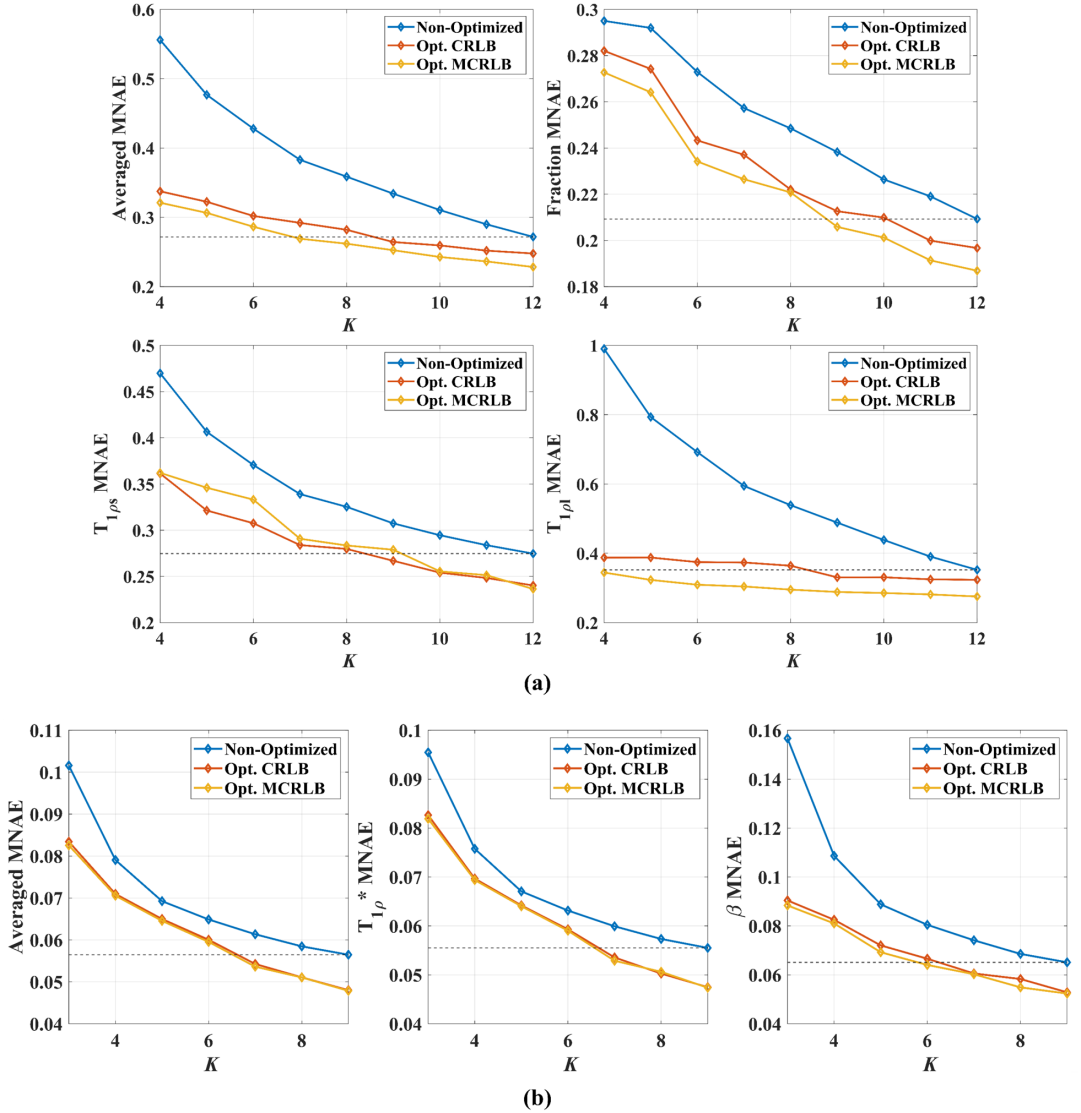
A weighted average of MNAE (**a**) for estimated bi-exponential parameters, MNAE for the fraction, short T_1ρ_, and long T_1ρ_ versus *K*. The optimized sampling schedules resulted in lower errors for the entire range, and the MCRLB optimized schedule was the top performer. Interestingly, the CRLB schedule performed better for the estimation of long T_1ρ_. This is probably due to the longer TSLs employed. In (**b**) Weighted average of MNAE for estimated stretched-exponential parameters, MNAE for the T_1ρ_*, and β versus *K*. Errors are much lower than for the bi-exponential model, and so is the gain over the non-optimized schedule. The differences between CRLB and MCRLB schedules also become smaller. The black dashed line indicates the minimum error obtained using the non-optimized schedule. The optimized schedules can reach this line before the non-optimized schedules, leading to lower acquisition times.

Figure 1a shows the estimation error and standard deviation for each parameter of the bi-exponential model using different schedules and also a combined error for all parameters according to their weights. The optimized schedules not only reduce error for the same *K*, but in some cases, the error was lower even when smaller *K* are used.

Similarly, Fig. 1b shows the estimation error and standard deviation for the stretched-exponential model. Although the differences between optimized and non-optimized schedules are smaller than for the bi-exponential model, the optimized schedules are consistently better in terms of MNAE. Also, the MCRLB optimized schedules performed consistently better than the CRLB optimized schedules.

**Figure 2:**
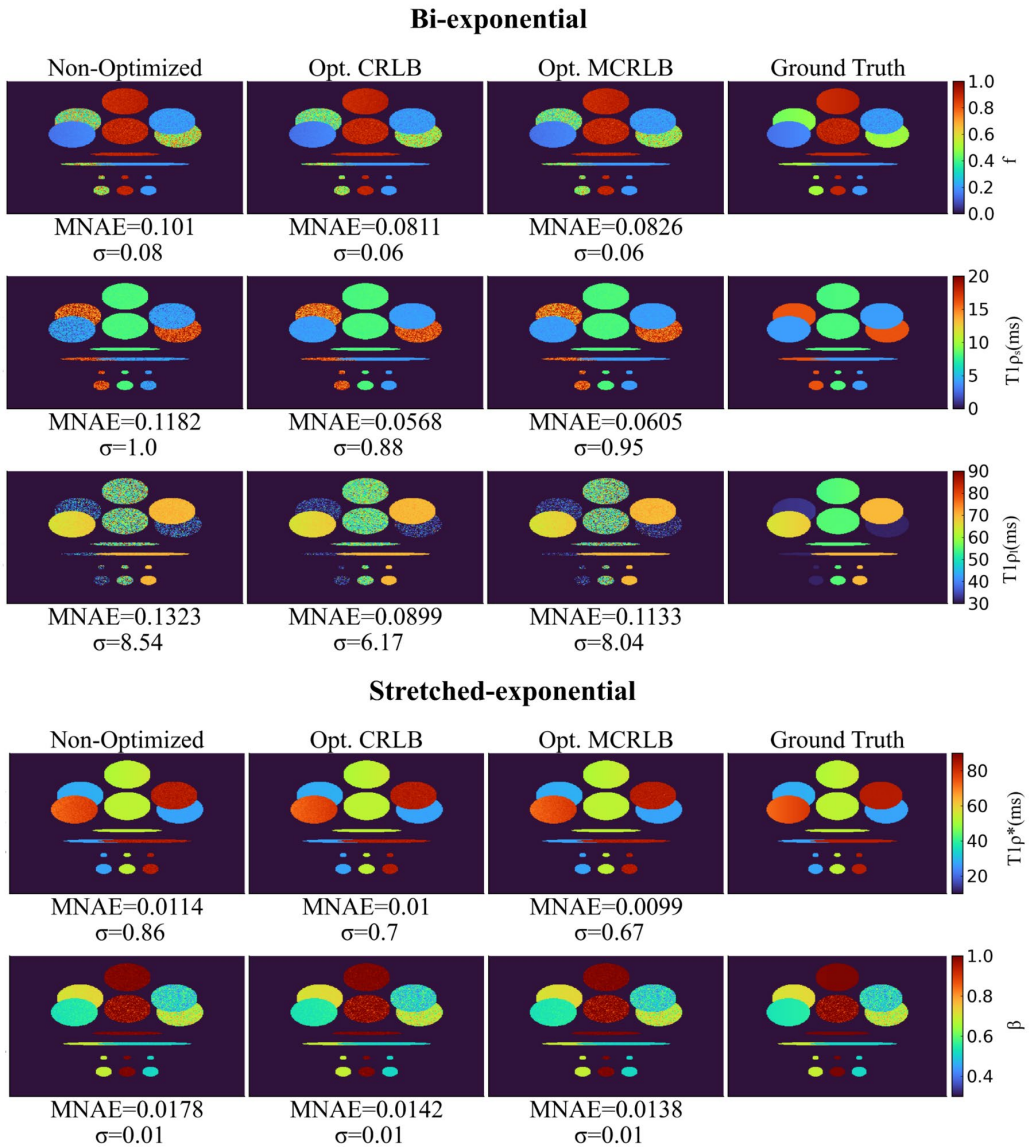
Parameter mapping for the bi- and stretched-exponential models with synthetic phantoms. The optimized schedules not only presented smaller MNAEs than the non-optimized ones but also presented a smaller standard deviation (σ) of the residue. Image generated in Matlab^27^.

Figure 2 illustrates results for parameter mapping in a synthetic phantom using *K* = 6. For the bi-exponential maps, the region where both short and long components are closer presented larger errors than the other regions. This is to be expected from the bi-exponential model. For the stretch-exponential maps, the errors are much lower and the differences between the maps are barely visible, still, the metrics show the improvement with the optimized schedules.

### Evaluation with model phantoms

For this experiment, we used only the schedules with *K* = 6. The reference maps for this experiment were obtained using all acquired TSLs for the 6 schedules used, which resulted in 19 TSLs due to common TSLs between schedules. The acquired TSLs are: 0.5, 0.5, 2.2, 5, 6, 9, 11, 11, 12, 17, 21, 22, 22, 24, 24, 33, 44, 55, 55 ms. The eggs were scanned whole, but the egg whites were not mapped for two reasons: much longer decays than is expected in knee cartilage, and predominantly mono-exponential behavior. From the total of 6 eggs, 2 were raw, 2 were soft-boiled, and 2 were hard-boiled. This way we can analyze a wider range of the parameters.

**Figure 3:**
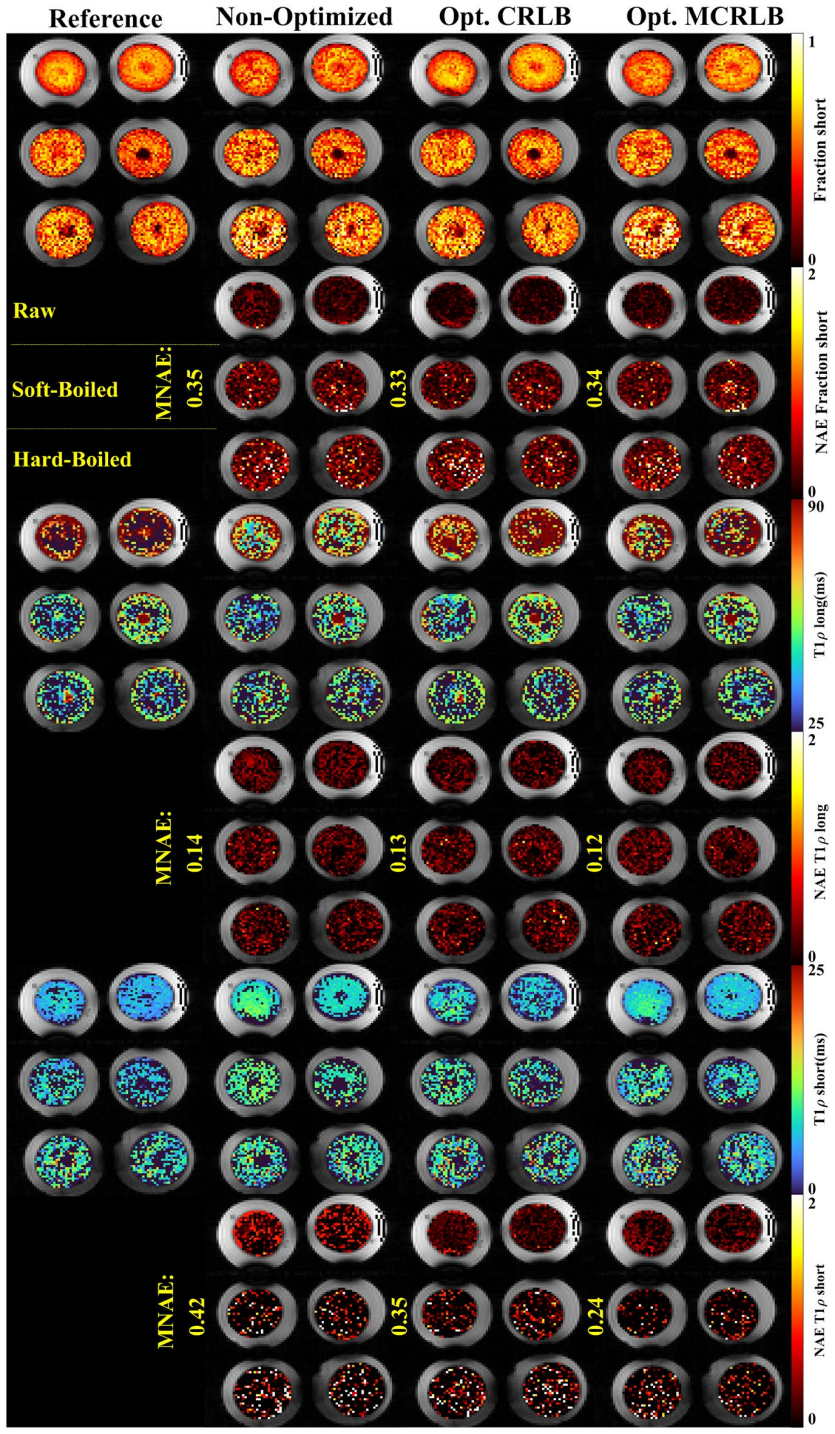
Bi-exponential parameter maps for the egg yolk phantom, the eggs are organized in rows as raw, soft-boiled, and hard-boiled, top-to-bottom order. The parameters fraction, long and short T_1ρ_, along with the voxel-wise Normalized Absolute Error (NAE) for each parameter. Image created using Matlab^27^ and Inkscape^28^.

**Figure 4:**
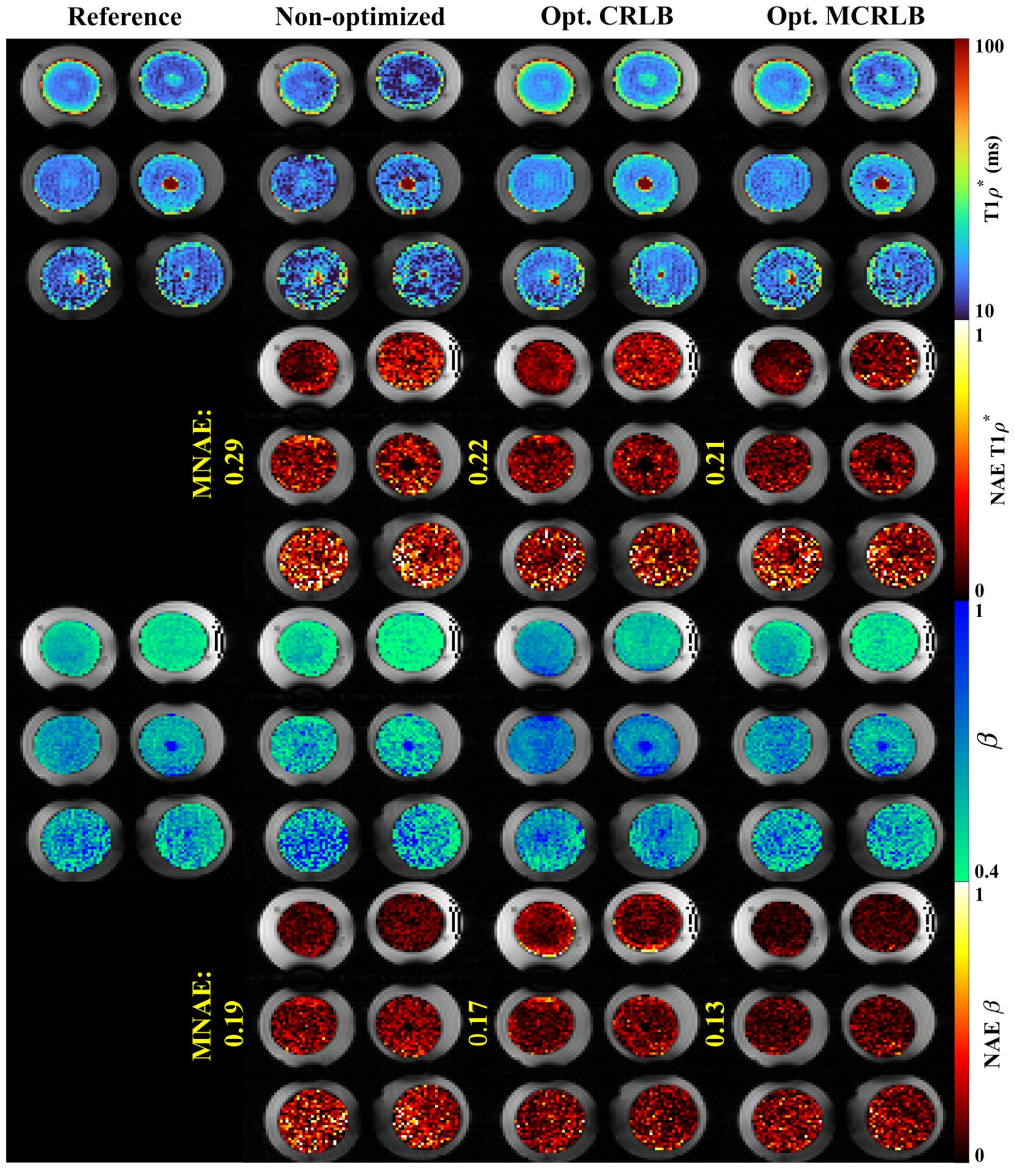
Stretched-exponential parameter maps for the egg yolk phantom. The parameters T_1ρ_*, and β, along with the voxel-wise Normalized Absolute Error (NAE) for each parameter. Image created using Matlab^27^ and Inkscape^28^.

Figure 3 shows the bi-exponential maps and the error maps. We see the biggest improvements for the short T_1ρ_ maps, especially for the MCRLB optimized schedule where the MNAE is close to half that of the non-optimized schedule. Figure 4 shows the stretched-exponential maps obtained with the different schedules and their error maps. The errors are smaller for the raw eggs due to higher SNR when compared with the hard-boiled eggs. We see a higher error in the β-map for the CRLB optimized schedule when compared to the MCRLB optimized one. This is probably due to the MCRLB not being dominated by the larger error.

These results are summarized in Table 3, optimized schedules performed better than non-optimized schedule in all metrics for both methods. Figure 7 top row shows the non-monoexponential relaxation in the egg yolks, which results in a lower R^2^ for the mono-exponential fitting when compared to the bi- or stretched-exponential fittings. When comparing the AICc of the multi-exponential models against the mono-exponential for each voxel in the reference, 59.2% of the voxels were better fitted with the stretched-exponential model and 21.1% with the bi-exponential model, the remaining 19.7% were considered mono-exponential.

**Table 3:** Results for the egg yolk and in-vivo knee mapping.

Schedules	Eggs				In-vivo			
	Bi-exponential		Stretched-exponential		Bi-exponential		Stretched-exponential	
	Av. MNAE	R^2^	Av. MNAE	R^2^	Av. MNAE	R^2^	Av. MNAE	R^2^
Non-optimized	0.47	0.74	0.28	0.80	0.471	0.533	0.047	0.876
CRLB	0.39	0.87	0.24	0.92	0.308	0.651	0.040	0.901
MCRLB	0.36	0.74	0.23	0.96	0.317	0.869	0.039	0.908

Average MNAE and R^2^ values for the optimized schedules are better than those obtained with the Non-optimized schedules.

### Evaluation with in-vivo knee cartilage

For in-vivo experiments, we recruited three volunteers to undergo the approximately 1-h scan, in which 19 TSLs were acquired. Same as for the model phantom, the 19 TSLs correspond to the TSLs acquired for the 6 different schedules. Due to the long scanning time, translational motion correction was applied to the data to mitigate the related errors. The cartilage was manually segmented from sagittal images of both medial and lateral slices of the knees.

**Figure 5:**
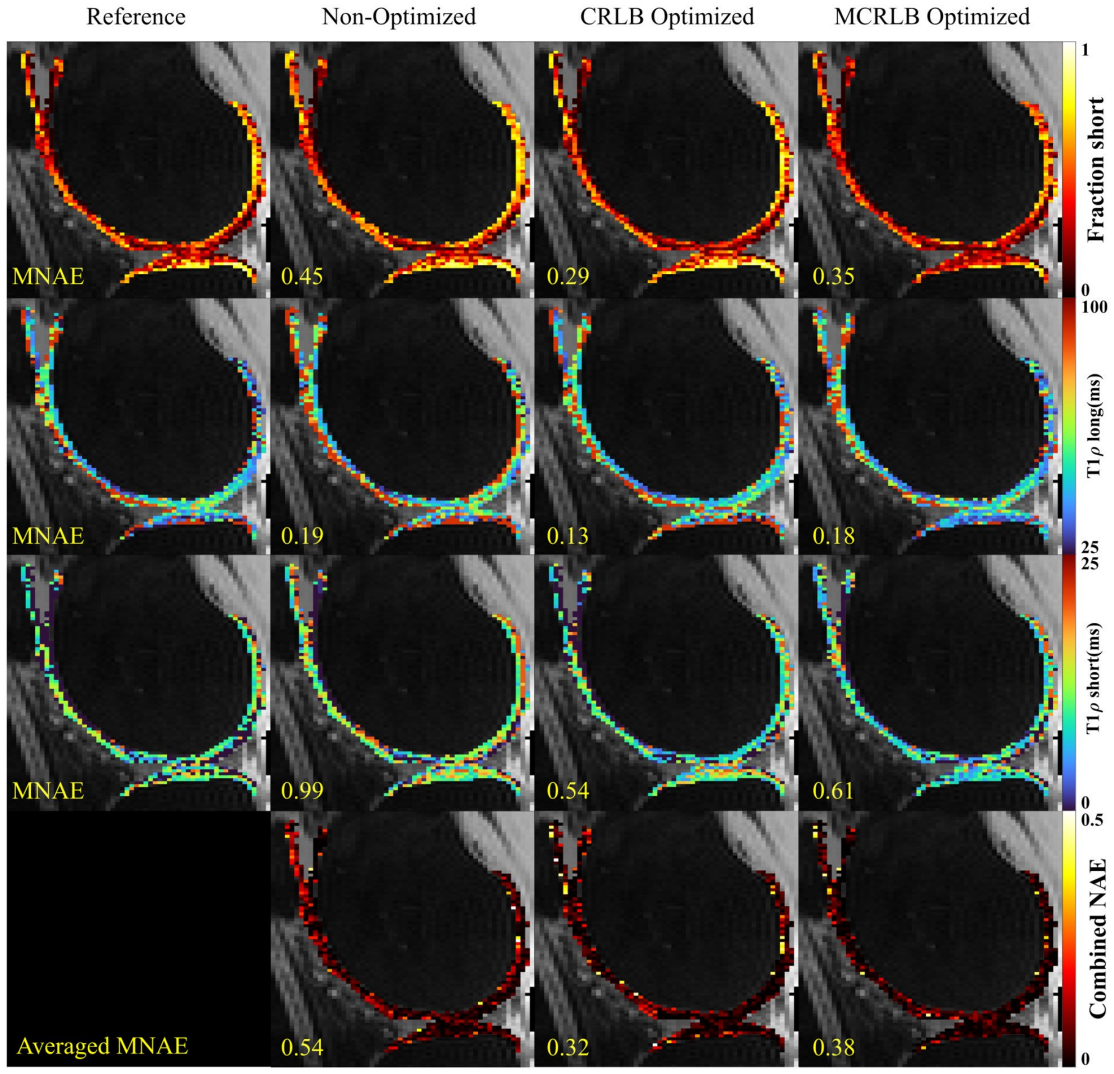
Parameter maps for the bi-exponential model fitted using the three different sampling schedules on the first volunteer dataset. The combined NAE uses the weighting vector used in the optimization to average the voxel-wise over different parameters. Image created using Matlab^27^ and Inkscape^28^.

**Figure 6:**
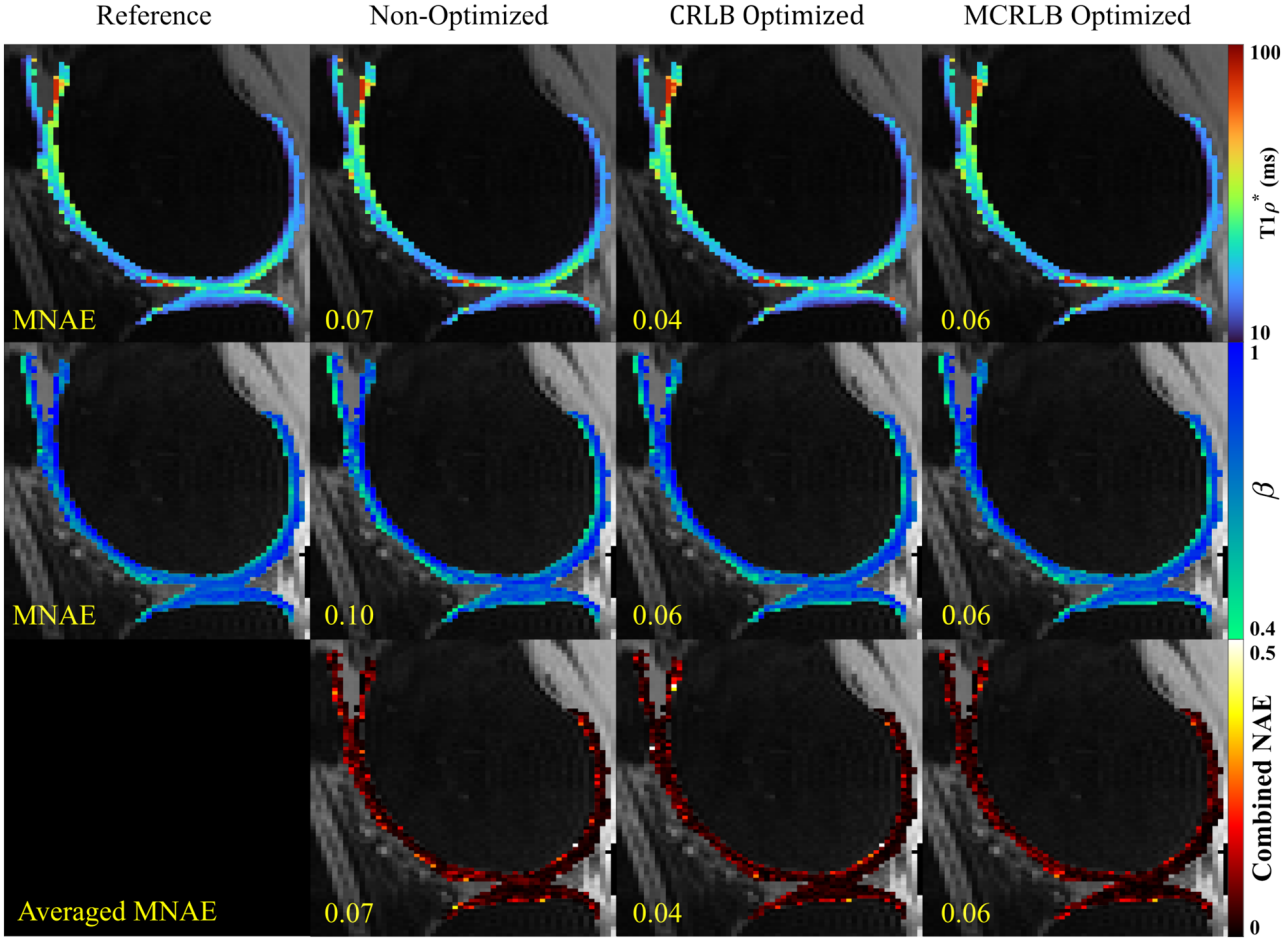
Parameter maps for the stretched-exponential model were fitted using the three different sampling schedules on the first volunteer dataset. The maps look very similar, but the combined NAE maps highlight the differences between them. Image created using Matlab^27^ and Inkscape^28^.

Figures 5 and 6 show the reference and estimated maps from a lateral slice of the first volunteer, using the bi-exponential and the stretched-exponential models, respectively. Also included are the combined NAE maps, these maps show the weighted average of the NAE as defined in Eq. (13). The combined values of MNAE and R^2^ for the three volunteers are summarized in Table 3. Again, the AICc comparison shows that most voxels are better fitted with the stretched-exponential model with 54.2% of the voxels. The voxels better fitted with the bi-exponential model accounted for 38.4% of the total.

**Figure 7:**
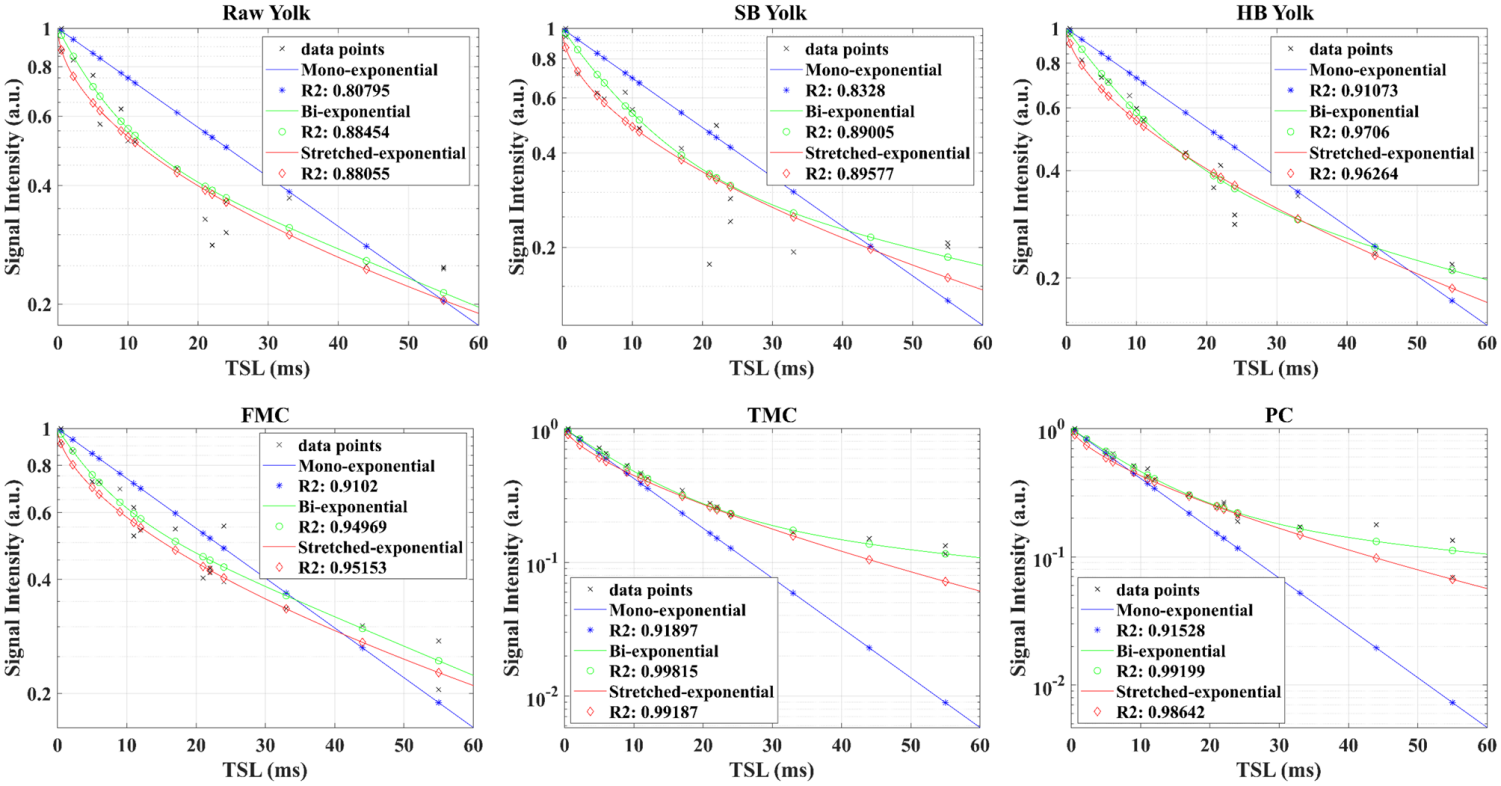
Fitting on representative pixels of raw, soft-boiled (SB), and hard-boiled (HB) yolks, and, of three compartments of knee cartilage: Femorl Medial Cartilage (FMC), Tibial Medial Cartilage (TMC), and Patellar Cartilage (PC). The y-axis is the normalized signal amplitude in the log-scale.

Figure 7 bottom row shows the non-monoexponential relaxation found in different cartilage compartments in the knee. We saw no significant difference in the number of voxels with non-monoexponential relaxation between compartments, but it was present in every compartment.

## Discussion

The optimized TSL schedules presented are capable of reducing acquisition time and improving fitting precision simultaneously. The reduction in acquisition time for the bi-exponential model is approximately 42% or 16.5 min. This means a reduction of 5 TSLs, as shown in Fig. 1 when compared to a reduction of only 2 TSLs for mono-exponential fitting^12^, this is a surprising result. For the stretched-exponential model, the reduction was approximately 22% or 6.6 min, similar to that of the mono-exponential fitting^12^. These reductions are the direct consequence of using fewer TSLs. The optimized TSL schedules also improve parameter accuracy when compared to a non-optimized schedule with the same number of TSLs. In some cases, it is possible to avoid the trade-off between reduced acquisition time and parameter accuracy, as an optimized schedule with fewer TSLs can be faster and achieve better accuracy. The use of accelerated sequences combined with Compressive Sensing^36^ might further improve these results. Another benefit to this method is that by reducing the number of acquired TSLs, one can use longer sequences or averaging to improve SNR. As the schedule optimization is done over the expected distribution of parameters for the object, performance can decrease if the object has a distribution different from the expected. This can be seen in the in-vivo experiment, in which the MCRLB-optimized schedule for the bi-exponential model performed slightly worse than the CRLB-optimized schedule for the same model. This probably happened because the scanned knees had more voxels with longer T_1ρ_ than was expected and as the CRLB-optimized schedule has longer TSLs, it was better suited for the fitting. Similar results were reported by Yuan et al.^11^ for mono-exponential fitting of T_1ρ_ relaxation. In that study, it was shown that a schedule with higher TSLs performs better for larger T_1ρ_ values than a similar schedule with lower TSLs. They also showed that the schedule with lower TSLs performed much better for lower T_1ρ_ values, obtaining a lower deviation over the whole range. This is consistent with our Monte Carlo simulations that show overall lower errors for the MCRLB optimized schedules.

Instead of optimizing the mean, we could have optimized for the worst-case in the expected distribution as in^13–15^ Optimization of the worst-case means that the optimal schedule’s error will have an upper-bound, but it comes with the cost of increasing the mean error across the distribution^37^. But under practical situations, with outliers and non-translational motion, these approaches may perform better.

The results obtained with the egg yolk model phantoms demonstrate the non-monoexponential relaxation that can be better fitted by multi-exponential models, as previously shown^25,26^. Although the voxels represented in Fig. 7 shows better fitting with the bi-exponential model, both the bi- and the stretched-exponential models performed similarly in terms of R^2^, as evidenced in Table 3. The results with egg yolks also show the effect of having a larger than expected T_1ρ_. Our optimization considered values of up to 90 ms, while higher values were shown in other work^26^. Indeed, the fitting for the raw and soft-boiled yolks is worse, in terms of R^2^, than the error for the hard-boiled yolk. This is in part due to our optimization considering a smaller interval, but also because the TSLs are limited to 55 ms due to SNR. This limitation of TSL has an impact on the fitting of longer relaxation times that will affect both optimized and non-optimized schedules.

The results for in-vivo knee cartilage also show that the majority of voxels were better fitted with the stretched-exponential model rather than the bi- or the mono-exponential models, following the AICc analysis. Similar results were reported^35^ for T_1ρ_ in the liver, where the majority of the voxels were better fitted by the stretched-exponential model. This better fitting does not mean that the stretched-exponential model is more useful, although recent works^23^ correlate the parameter β with a decrease in GAG and water content in inter-vertebral discs, and increased contrast in T_1ρ_ maps, the same has not been demonstrated for knee cartilage.

Since there is no ground truth to the model phantom and in-vivo cartilage maps, we made use of all the TSLs acquired to build a reference. This way, each schedule can be considered as a subset of the reference TSLs. With this, we try to minimize the effects of any bias introduced in our comparison between schedules. Also, the use of a higher number of TSLs makes the reference more robust, since the difference in errors obtained with the schedule goes down with size. Using synthetic data for the case where K = 19, we compared the difference between non-optimized and optimized schedules for the bi-exponential model parameter estimation. Using the same 19 TSLs for model phantoms and in-vivo cartilage and a uniformly spaced schedule: 0.5, 3.5, 7, 10, 13, 16, 19, 22, 25, 28, 31, 34, 37, 40, 43, 45, 49, 52, 55 ms. The difference in MNAE obtained with both schedules was 0.00275.

Another limitation of this approach is the fact that the NLS estimator is biased. As the CRLB is a variance bound for unbiased estimators, optimizing the schedules based on the CRLB can have a lower-than-expected impact. Since the biased CRLB requires the bias gradient to be calculated, the process of optimization would require the estimation of this gradient for every combination of TSLs. This can be simplified by direct estimation of bias and variance of the estimator for every schedule which could lead to the minimization of MSE, as done by Zibetti et al.^12^ with the MSF approach. That resulted in a similar performance to the simpler minimization of the CRLB and MCRLB, but with a computational time of up to 100 times greater. As the computational time is even longer for bi- and stretched-exponential models because of the increased complexity of the models, we decided not to use MSF approaches or biased CRLB in this study.

The approach investigated is not limited to T_1ρ_ mapping, but can be used in other quantitative techniques such as T_1_ and T_2_ mapping^13,14,16^, as well as MRF^17,18,38,39^. As far as we know, there is no other study comparing CRLB optimized schedules for bi- and stretched-exponential models. Kratzer et al.^38^ used the CRLB as criteria to optimize a Sodium MRF sequence for T_1_ and T_2_ mapping. However, they simplified the T_1_ relaxation as a mono-exponential and optimized only for that, ignoring the T_2_ bi-exponentially relaxed signal.

Future studies include the use of variational networks^40,41^ proved to be capable of reconstructing images from undersampled k-space data. The use of this kind of network along with another network responsible for data fitting, such as Recurrent Inference Machines (RIM)^42^, can be used to form an end-to-end network for quantitative parametric mapping. Such an approach might enable the combined optimization of TSL scheduling along with the network parameters.

## Conclusions

In this study, two different optimization criteria for choosing the TSLs for T_1ρ_ mapping were compared for two multi-exponential models. According to our results in synthetic data, model phantoms, and healthy volunteers, both the CRLB and the MCRLB optimized schedules outperform the simple linearly spaced schedule. The optimization of sampling schedules based on the exact minimization using the CRLB is a procedure that increases in time according to the number of TSLs acquired and the complexity of the model, but it will still be faster than methods such as MSF. The optimized TSLs with these methods allowed for improved results even when fewer TSLs are used, when compared to a non-optimized sampling schedule.

## Supplementary Information

Supplementary Information.

## Data Availability

The datasets generated during and/or analyzed during the current study are available from the corresponding author upon reasonable request.
