# Supplementary material for: Optimization of spin-lock times for T1ρ mapping of human knee cartilage with bi- and stretched-exponential models
Source: Sci Rep. 2022 Oct 7;12:16829. doi: 10.1038/s41598-022-21269-2 (PMC9546896; doi:10.1038/s41598-022-21269-2)
Supplement: Supplementary file 1 — Supplementary Information. [file 41598_2022_21269_MOESM1_ESM.pdf]

Optimization of Spin-Lock Times for $T_{1\rho}$ Mapping of Human Knee Cartilage with Bi- and Stretched-exponential Models

Hector L. de Moura*[1], Rajiv G. Menon[1], Marcelo V. W. Zibetti[1], and Ravinder R. Regatte[1]

[1] Center for Biomedical Imaging, Department of Radiology, New York University Grossman School of Medicine, New York, NY, USA

[*] Hector.LisedeMoura@nyulangone.org

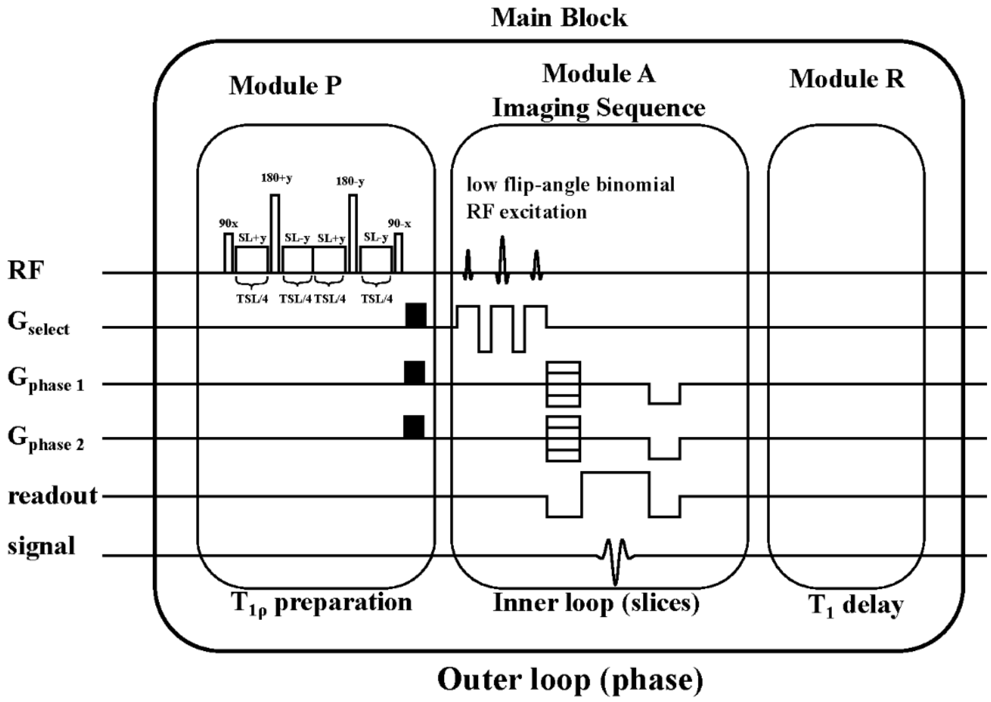

Figure S1. Pulse sequence for 3D $T_{1\rho}$-weighted acquisition. The sequence is composed of a preparation module P, an acquisition module A, and a magnetization restoration module R. The modules are repeated to acquire the full k-space data.

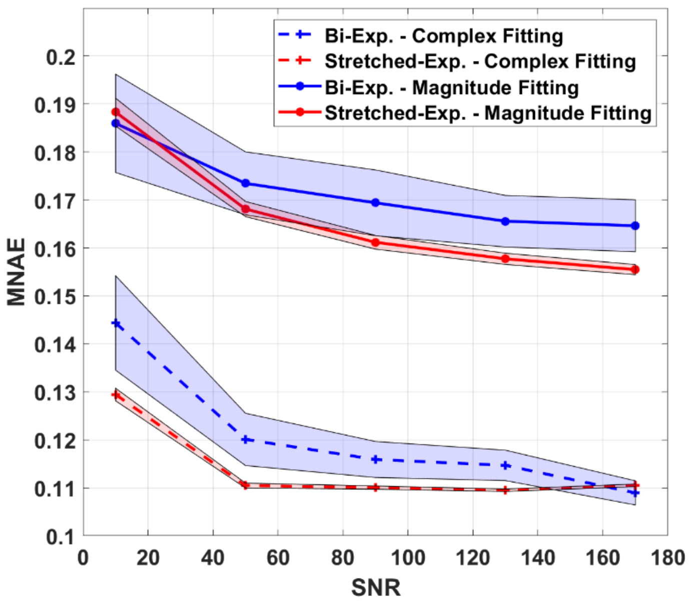

Figure S2. Comparison of estimation error using magnitude-only versus complex fitting for different levels of SNR. The central lines represent the mean value for one hundred iterations and the bands represent the standard deviation. The complex fitting shows smaller errors across the entire range for both models tested.
